# “No matter how hot it is, you just have to do the work”: Examining farmworkers’ experiences with heat and climate change in Idaho

**DOI:** 10.1016/j.joclim.2024.100300

**Published:** 2024-01-23

**Authors:** Carly Hyland, Delmy Flores, Grace Augusto, Irene Ruiz, Marielena Vega, Rulon Wood

**Affiliations:** a Division of Environmental Health Sciences, School of Public Health, University of California Berkeley, Berkeley, CA, USA; b School of Public and Population Health, Boise State University, Boise, ID USA; c Idaho Organization of Resource Councils, Boise, ID USA; d Department of Theatre, Film, and Creative Writing, Boise State University, Boise, ID USA

**Keywords:** Farmworker, Heat, Wildfire smoke, Climate change, Environment, Disparities

## Abstract

**Introduction:**

Farmworkers are one of the populations most vulnerable to climate change. In addition to disproportionate exposure to climate-intensified hazards such as extreme heat and wildfire smoke, many farmworkers face situational and psychosocial stressors that also impact their health.

**Methods:**

We conducted open-ended interviews with 16 farmworkers in Southcentral Idaho during June-August 2023 as part of a digital storytelling project in partnership with the Idaho Organization of Resource Councils (IORC). Interviews assessed participants’ experiences with climate change, perceived impacts on their health, and strategies and barriers to protecting themselves. Bilingual (English/Spanish) study staff translated and transcribed all video recordings verbatim, and we abstracted representative quotes from interviews.

**Results:**

Participants primarily discussed the health impacts of heat, recounting personal experiences and stories of other farmworkers feeling dizzy, wanting to vomit, or passing out from occupational heat stress. Despite these experiences, they reported feeling like they needed to keep working regardless of the conditions in order to get paid and support their family. Experiences with climate change were underscored by participants’ discussions of co-exposure to situational and psychosocial stressors such as lack of access to healthcare, poor housing without air conditioning, and racism, discrimination, and fear of family separation.

**Discussion:**

This project highlights farmworkers’ first-hand experiences regarding climate change and underscores the interconnected impacts of occupational, environmental, and psychosocial stressors on their health and wellbeing. These findings emphasize the importance of shifting the burden of climate resiliency from individual farmworkers to systemic workplace, residential, and community interventions.

## Highlights

• Interviews with farmworkers about experiences with heat and climate change.
• Symptoms of heat included dizziness, headache, fainting, wanting to vomit.
• Insufficient duration and frequency of paid breaks to protect from heat.
• Occupational heat exposure exacerbated by situational and structural inequities.
• Interventions needed to protect farmworkers from heat and climate change.

## 1 Introduction

Farmworkers are disproportionately impacted by many climate-intensified exposures [1,2] and will continue to be one of the populations most vulnerable to global climate change. Specifically, farmworkers face numerous environmental and occupational threats to their health that will continue to worsen with climate change, including exposure to pesticides, extreme heat, wildfire smoke and other air pollutants, and biological agents such as viruses and bacteria [3]. Latinx farmworkers, who represent over 80 % of the agricultural workforce in the U.S. [4], are particularly vulnerable to the impacts of these exposures due to situational and structural factors such as limited control over workplace exposures and added barriers to health protection, including limited economic, health, and social resources [3,[5], [6], [7]]. While quantitative studies have begun to characterize these climate-intensified stressors, data gaps exist regarding farmworkers’ own perceptions and experiences with climate change, including strategies to protect themselves and barriers to protection.

Despite being deemed “essential workers” during the Covid pandemic, farmworkers across the United States have reported feeling “invisible” [8,9] and “expendable” [10] in recent years. These feelings are supported in recent findings highlighting disconnects between consumers’ interest in food production systems and their knowledge regarding the processes required to get food to their table. For example, a nationwide survey commissioned by the U.S. Farmers & Ranchers Alliance in 2011 indicated that while nearly 70 % of consumers reported thinking about food production at least “somewhat often”, 72 % reported knowing “nothing” or “very little” about farming and ranching [11], underscoring the importance of elevating farmworkers’ perspectives and experiences to educate the general public about how their food is produced.

Data suggest that story-based media can shift social norms, values, and beliefs more effectively than fact-based messaging [12] and can also reach wider audiences. We conducted a digital storytelling project with Latinx farmworkers in which we aimed to (1) humanize this essential workforce, (2) provide safe spaces for farmworkers to share their experiences and build community, (3) understand farmworkers’ experiences with climate change, and (4) educate consumers regarding the processes required to produce our food. We have created various public-facing written and visual materials from this project; here, we report findings regarding participants’ experiences with extreme heat and climate change.

## 2 Methods

### 2.1 Recruitment and enrollment

We recruited a convenience sample of participants in partnership with farmworker-serving organizations including the Idaho Organization of Resource Councils (IORC) and Latinx Farmworkers of Southern Idaho, as well as through snowball sampling. Bilingual (English/Spanish) study staff approached potential participants, assessed their eligibility to participate (i.e., 18 years or older, spoke English or Spanish, currently working as agricultural worker in Idaho), and read a short recruitment script describing the background and goals of the project. If the individual was interested in participating, study staff read an Information Sheet that detailed the goals and procedures of the project and emphasized that participation was voluntary and that the individual could withdraw at any time. The Information Sheet also detailed various options for participation, including participating in a video recording, an audio recording without any video or images, or simply talking with us while a study member transcribed the conversation without any recording. If the participant selected to participate in a video and/or audio recording, we provided them the option to have their face and/or voice blurred to protect their anonymity.

We took various other steps to protect the anonymity and confidentiality of study participants. Notably, we did not collect any identifying or demographic information from the participants. We gave each participant a copy of the Information Sheet with a unique ID number and the Principal Investigator's contact information and indicated that they could contact us at any time if they wanted to withdraw from the project, and that we would not use any of their data. We labelled all recordings and transcriptions with the individual's anonymous participant ID that cannot be linked to any identifying information and stored all materials in a password-protected database in a locked lab at Boise State University. Each participant received a $25 gift card. All procedures were reviewed and approved by the Boise State Institutional Review Board.

### 2.2 Data collection and analysis

All data collection occurred in the participant's preferred language (English or Spanish). We conducted semi-structured interviews with 16 farmworkers in Southcentral Idaho from June-August 2023 using an interview guide with open-ended questions assessing participants’ experiences working in agriculture, the biggest challenges they faced as a farmworker, what they would like the public to know about their work, and their perceptions of the impacts of climate change on their work and overall health, including strategies and barriers to protecting themselves from heat. All participants elected to participate in the video recording. Two bilingual (English/Spanish) study staff translated and transcribed all video recordings verbatim, and we abstracted quotes to identify overarching themes related to participants’ experiences with heat and climate change.

## 3 Results

We identified six main themes regarding participants’ experiences with climate change and extreme heat, including: (1) personal experiences with heat, (2) other farmworkers’ experiences with heat, (3) strategies and barriers to protect from heat, (4) impacts of heat on the use of Personal Protective Equipment (PPE), and (5) experiences with other climate-intensified exposures (representative quotes shown in Table 1), as well as (6) situational and structural factors exacerbating the health impacts of occupational heat exposure (representative quotes shown in Table 2).

**Table 1:** Representative quotes regarding participants’ experiences with climate-intensified environmental and occupational exposures.

Theme	Representative Quotes
Personal experiences with heat	“We definitely noticed how much hotter the seasons just came to be… I mean, it'd be like 06:00 a.m. and you're already suffocating with the heat, especially when you were detasseling corn.”
	“I have had a headache and wanted to vomit because of the heat…you feel the pressure and the head hurts and, well, it makes you dizzy…”
	“When it is very hot, I experience a lot of lethargy. There are even times where I want to give up, but then I know work must be done, and I continue.”
	“I've seen it in the amount of just how hot it is every year and how it doesn't die down. It's like nine and it's still pretty much hot out there. And you're like, barely getting home from work or at night when you're trying to sleep, you've been in the heat all day long and then at night you're trying to cool down and it's still very hot.”
Other farmworkers’ experiences with heat	“I have heard incidents of heat, where they have gotten bad…The story is that they were working and it was very hot weather, then suddenly he became weak and fainted.”
	“I have heard of incidents that have happened because of the heat. I have heard specifically of sickness, but I have heard how many people become anxious, start losing their focus and orientation. Also, they start developing pimples and/or develop some sort of allergies in their body.”
	“I have heard about an incident of someone getting sick because of the heat…There are stories are about people that experience so much heat, they have fainted or have gotten dizzy, and cannot continue working, until they are hydrated again, or took a longer break.”
Strategies and behaviors to protect from heat	“Besides drinking water on those hot days, I take a small break, and try to go to a cooler area. However, there are just a few trees in the area to get shade and get cooler.”
	“The opportunities to cool off are very low because very often are you in a place where there's a tree nearby but it's not enough for everyone to sit by, right? The breaks, they're not that long. It'd be great if we had more breaks that were paid…maybe you get, like, a paid 15 min break, and then you get an unpaid 30 min break. And a lot of people, they're like, they would want to not take breaks to get that money. But it'd be great if we had opportunities to take breaks that were paid.”
	“When it is hot our job is a lot harder. It is just insanely hot. No matter how hot it is, you just have to do the work… I drink a lot of water when it is hot to combat the heat. I also wear a long sleeve shirt, a hat, and hydrate to overcome it.”
	“…with a break that they give us of 15 min, we recover a little, we hydrate ourselves. In fact, we each carry frozen water to stay hydrated and not have more serious consequences.”
	“I have the handkerchief. We put on this scarf like this because when it's very hot, you sweat and this scarf gets wet. And that keeps you fresh…the day I forget it and don't wear it, I feel like I'm around a bonfire that burns my face”
	“The only thing that you can do is have more water breaks but not everyone gives you more water breaks.”
Impacts of Heat on PPE Use	“We have to continue to be fully clothed because of other elements are out there that can harm us like pesticides…also just covering yourself because you don't want to deal with skin cancer… When I worked in the field, we did not we did not take off our long-sleeved shirts…no matter how hot it got we stay fully clothed.”
	“When it is hot outside, I tend to wear the least PPE for the heat. I only use a sweater, because if I put on more layers, I start sweating so much. I feel suffocated.”
	“The only protection I use in the heat is sunscreen. I also cover myself with a face covering when it's hot. Here in my skin, you can see the difference in my skin tone. I do have access to shade.”
Other Climate-Intensified Exposures - Wildfires	It's so hard to breathe. I'm like, but there's still some people outside, like, working in that freaking smoke because they have to and they cover their faces with a bandana, but that does little to nothing. And you breathe out all day and it's just hard on your lungs. So, when you get home, not only are you achy from your joints from doing this position all day, carrying stuff on you or bending down, you have all this pressure in your lungs from having to breathe really bad air and just overworking yourself.

**Table 2:** Representative quotes regarding participants’ experiences with situational factors and psychosocial stressors exacerbating the impacts of climate-intensified exposures.

Theme	Representative Quotes
Lack of regulation or ability to be involved in policy to protect from climate-intensified exposures	“The usual schedule for seasons has changed so much where people start working earlier, start working later, people are having a hard time adjusting to what these changes and seasons are because of changes in climate…We've seen that there's a lot more wildfires. We've seen that it's been getting hotter, that the heat is lasting for longer throughout the day. And that's what's challenging because there's already no rules or standards set in place to protect farm workers. That we're just expecting farm workers to just go with the flow. That as our climate and as the world is getting hotter that we're just expected to kind of fall down because there have been no changes, no advocacy towards like, this is what you should do, because it is getting hotter. Maybe we should give an hour, 30 min break for folks, right? That we're just expected to follow through with what's going on and still expected to do the work that we're doing but without getting any kind of help, assistance or aid at all, that we're just supposed to continue to do what we're doing and have that not change even though the world around us is changing.”
	“I know a lot of people in the farm worker community who would love to be at the table and share stories and do listening sessions and be part of policy making, but they can't because they cannot afford to do so. They cannot afford to take time off work. And whatever time they do have, they try to enjoy it at home, to be resting and be with their families.”
Lack of control to choose whether to engage in agricultural work	“It wasn't really a choice I think a lot of us who are from workers do it because we need to support our families and we need to put food on our table”
	“I was 10 when I started working and I know that's young but and a lot of farms you know even kids start working at a young age.”
	“I've done farm work since I as long as I can remember in some capacity. My parents didn't have enough money to pay for a babysitter and they didn't really trust anyone in the area.”
Lack of healthcare	“My parents used to not go to the doctor or even get checkups visits or even vaccinations because it all came out of pocket for them.”
	“It'd be great if we had access to health insurance, so when we felt sick, instead of complaining and being you know what, I've had a stomachache for days. Let me go check it out. It's like I had a stomachache for days, but I'm still here. Yeah, I might look really bad, but I'm still here because I need the money. I can't afford the doctor visit. The doctor visit is going to be more costly than me coming here and working and possibly fainting, right? So, it'd be great to have that, and especially for mothers, it'd be so great to have that paid maternity leave.”
Fear of law enforcement and family separation	“My parents were undocumented at the time. Asking them for a ride was a no go. Asking our godparents or other family members for rides was a no go because they were also undocumented and already scared of driving to work. Why would we put them in danger of getting stopped by police being undocumented?”
	“I always grew up with that fear of when a police officer is passing you by the road, be calm, look straight ahead, make sure everyone has their seatbelts. Even though we always did, we always kind of held our breath as a police car would pass by because we just didn't want to be recognized. We did not want to be noticeable because our parents were undocumented. And we live with that fear that we would be separated from our family. And that's something that wasn't fair and shouldn't have been something for a kid to kind of have to go through.”
	“There's so much that it means to be a farm worker outside from picking and doing what we think about with the job…it means driving in fear even though you shouldn't, right? You're a good driver and you might want to have a driver's license, but your local state doesn't want to give you that.”
Work schedule	“The difference between hours from Mexico and here, is that in Mexico I work 8 h only, the most 10 h. Here I work 12 h, 14 h, even 16 h. Sixteen hours without stopping.”
	“My routine of the day is that you wake up at 4 am in the morning and then do breakfast. By 6 am we start working. We work until 4:30 pm. Then after that time, we can work extra hours if we want. Sometimes we stay working until 8 or 9 pm at night. The latest I have stayed working has been 10 pm.”
Disproportionate impacts among women	“So, for women, it's so easy for us not to say anything, not to complain, because we're out there alone and we want to keep our jobs and we'll handle whatever we have to handle in order to keep those jobs and feed our family, and that's not okay.”
	“A lot of mothers who I've seen are pregnant, working in the fields. I had someone, like, picking apples next to my parents…during COVID. And she's like, I'm a single mom. I'm not going to have any maternity leave. I'm going to be out of work for at least one or two months while I recover, and then I'm going to go back. And women are forced to not even have time or space to recover after giving birth because they have to go back, right? And they don't have time to really bond with their kid or really have to take care of themselves.”

### 3.1 Personal experiences with heat

Participants reported various health symptoms from working in heat, including dizziness, headaches, feeling lethargic, and wanting to vomit. One participant noted how each season seemed to be getting hotter, indicating that by 6 A.M. it feels like “you're already suffocating with the heat”. One participant also discussed how it is difficult to cool down when they return from work and when they are trying to sleep because it is still very hot. Despite these impacts, one participant noted that even when they want to give up, they know “the work must be done and [they] continue”.

### 3.2 Other farmworkers’ experiences with heat

Multiple participants reported observing a coworker or hearing of another farmworker having an acute health event as a result of occupational heat exposure, including losing focus and orientation, becoming dizzy, fainting, and developing allergies. Similar to participants’ descriptions of their own responses in which they reported continuing to work regardless of the conditions, one participant reported hearing stories of people who had become dizzy or fainted and who couldn't continue working until they “hydrated again, or took a longer break”.

### 3.3 Strategies and barriers to protect from heat

The primary strategies participants reported to cool down and protect themselves from heat included carrying cold water, trying to find shade, and wearing a bandana, hat, or long clothes. For example, one participant reported that they wear a handkerchief, and when it gets really hot, they sweat a lot and the handkerchief gets wet and this keeps them cooler. Participants consistently reported that the frequency and duration of breaks were insufficient to help them cool down. Specifically, two participants reported getting 15-minute breaks where they can “recover a little”, with another indicating that additional water breaks could help, but “not everyone gives you more water breaks”. One participant indicated that they could take additional unpaid breaks, however many people do not because they need the money. These findings are underscored by participants reporting working extremely long shifts of 10–16 h (Table 2). Participants also reported that shade can help them cool down, but there is rarely enough shade for everyone.

### 3.4 Impacts of heat on PPE use

Responses from participants highlighted a tension in the use of Personal Protection Equipment (PPE) to protect from the sun and heat versus pesticides. While one participant noted that they continue to wear PPE regardless of how hot it is in to protect themselves against pesticides and skin cancer, another reported that they wear less PPE when it is very hot, indicating the feel “suffocated” if they wear additional layers in the heat.

### 3.5 Other climate-intensified exposures – wildfire

One participant discussed their experiences with wildfire smoke as another climate-intensified occupational exposure they are concerned about. This participant noted that farmworkers continue to work during wildfire events and while some may cover their faces with a bandana this does “little to nothing”. This participant discussed how damaging conducting intense physical labor during wildfire events can be on their lungs and overall health, noting that even after work they feel “all this pressure in your lungs from having to breathe really bad air and just overworking yourself”.

### 3.6 Situational and structural factors exacerbating impacts of heat

Participants identified and discussed various situational and structural factors that exacerbate the impacts of these climate-intensified occupational exposures, including fear of law enforcement and family separation and a lack of regulation, control in deciding to engage in farm work (including as a child), access to healthcare, and ability to be involved in advocacy efforts. Notably, we did not ask specific questions on these “non-chemical” exposures, and these themes emerged without prompting from the study staff.

One participant discussed how they feel there is already a lack of regulations to protect farmworkers, and no additional protections have been put in place even as climate change continues to worsen with increased heat and more wildfires. A participant also talked about how many farmworkers would like to be engaged in policymaking but are excluded from this process and would not be able to afford to do so if invited because they cannot afford to miss work. Some individuals also discussed the lack of control they had in choosing whether or not to engage in agricultural work as an occupation, even as a child, and that it is necessary “because we need to support our family and we need to put food on the table”.

Participants also discussed stressors such as lack of access to healthcare. Specifically, one participant noted that they often do not go to the doctor, even if they have felt ill for days, because they do not have health insurance and it would be more costly than going to work. Themes regarding fear of law enforcement and family separation also emerged. One participant noted that even as a child they lived with fear that they would be separated from their family, and multiple participants discussed fear of the police in relation to being racially profiled while driving and not being able to obtain a driver's license.

One final theme that emerged is the potential disproportionate impacts and vulnerability of women farmworkers. One participant alluded to the additional difficulties women face in advocating for themselves for fear of losing their job, and another discussed her observations of pregnant farmworkers continuing to work in the fields, including during Covid. This participant highlighted the lack of paid leave or care during the perinatal period and that women farmworkers often do not have time to recover or bond with their child before needing to return to work.

Finally, one participant shared the following quote that summarizes many of the experiences we heard regarding the impacts of chemical and non-chemical stressors on their health and wellbeing:

“There's so much that it means to be a farm worker outside from picking and doing what we think about with the job. It means taking on the heat. It means taking out the smoke. It means trying to find work wherever possible, no matter what season it is. It means driving in fear even though you shouldn't, right? You're a good driver and you might want to have a driver's license, but your local state doesn't want to give you that. It means having to take a lot of unnecessary comments and unnecessary emotional and mental damage by people who are supposed to be looking out for you. Your crew bosses, the farmers and employers. You don't realize how cruel people can be in those positions because you are a worker. They think it's okay to yell at you, it's okay for them to tell you do this to overwork you. And if you want to take a bite of an apple, take a handful of cherries that you help pick. It's like being reprimanded for even wanting to take a little bit because you're taking away from the farm, right? Even though you've already given so much. So, there's so much to being a farm worker, right”

## 4 Discussion

Climate change has led to a rapid increase in exposures such as extreme heat and wildfire smoke, the effects of which have been and will continue to be borne disproportionately by farmworkers. In addition to the intense physical nature of their occupation, which is often outdoors with little access to shade, many farmworkers are co-exposed to psychosocial stressors that exacerbate the impact of climate-intensified exposures, including food insecurity, poor housing conditions, racism and discrimination, lack of social support, and barriers to accessing healthcare [3,[5], [6], [7]]. This project highlighted some of the impacts of these exposures on farmworkers’ health, and also illuminated the limited individual control that many farmworkers have over their occupational exposures or opportunities for protection. These findings underscore the need for systemic changes inside and outside of the workplace to improve climate resiliency in this essential population, as well as shifting the burden of protection from individual farmworkers to upstream interventions.

Farmworkers are at particularly high risk of Heat Related Illnesses (HRIs) due to factors such as performance of intense physical labor [13], [14], [15]. Notably, data suggest that farmworkers in the United States have 35 times the risk of heat-related mortality compared to the general workforce population [16]. Further, the Intergovernmental Panel on Climate Change (IPCC) has identified populations of low socioeconomic status at particular risk for HRIs due to factors such as inability to access healthcare and quality housing [17], factors that have been well documented among farmworkers [3]. In addition to limited control over workplace health and safety factors (e.g., access to water, shade, and rest), Latinx farmworkers experience multiple situational factors including poor housing conditions without air conditioning [14] and limited access to federal aid, legal assistance, and health programs that exacerbate the impacts of occupational exposure to heat and wildfire smoke [2]. This is underscored by overwhelming evidence that farmworkers’ exposure to wildfire smoke and heat are only going to continue to worsen in the coming decades. For example, the number of days agricultural workers spend in conditions exceeding heat safety standards is expected to double by 2050 [18], and data suggest wildfire-related air pollution may double to triple in the Pacific Northwest by the end of the century [19].

Participants in our study discussed various experiences with occupational heat exposure among themselves and other farmworkers, describing impacts on their health such as feeling dizzy, wanting to vomit, and hearing of others fainting or passing out due to the heat. One theme that emerged when discussing participants’ experiences with heat is how much hotter it appears to be getting each season, and how difficult it is to cool off or sleep at night due to the heat. These personal experiences are supported by mounting evidence of the increasing frequency, duration, and intensity of heatwaves, as well as historical trends in increasing daytime and nighttime temperatures [20,21]. In addition to impacts on sleep quality [22], as observed in this study, increasing nighttime temperatures may be particularly dangerous for populations without air conditioning, as this is a vital time for them to cool off and get reprieve from the heat. Emerging evidence suggests that higher nighttime temperatures contribute to heat-related mortality [22], [23], [24], even when controlling for daytime temperatures, underscoring the importance of populations working outdoors being able to get relief at night.

Many studies investigating interventions aimed at mitigating heat stress among agricultural workers have focused on behavioral changes, such as drinking more water or wearing cooling vests [25], [26], [27], [28]. However, individual-level interventions will likely be insufficient in preventing heat stress due to structural barriers farmworkers face [29]. Notably, in this and previous studies, farmworkers shared that they would often choose not to take additional breaks even if they were feeling unwell from the heat, as they would be unpaid and they feel pressure to make enough money to care for their family. Further, it is necessary to holistically examine potential unforeseen consequences and engage farmworkers in the development of interventions. For example, one of the most common recommendations to prevent occupational heat illness is to drink water, yet farmworkers have widely reported that they do not have consistent access to clean bathrooms and sometimes intentionally avoid hydrating. In previous work by our group, farmworkers have reported avoiding using the bathrooms because they are so dirty, or relieving themselves in the field; women, in particular, have reported ‘holding it’ until after work. These findings suggest that without engaging employers and labor contractors and developing workplace interventions (e.g., structured team breaks, access to cold water and electrolyte drinks, access to clean and safe bathrooms, workplace misting or shade canopies, team education on HRI signs, symptoms, and prevention strategies), efforts to prevent occupational heat illness and death may fall short.

Notably, while states like California have passed heat illness prevention regulations to protect outdoor workers, a recent data review suggests that heat-related mortalities among farmworkers in the state may be severely undercounted [30]. This report also suggested that heat-related morbidity and mortality may occur among farmworkers at temperatures below the regulatory threshold in which employers are required to provide water, rest, and shade, suggesting that current regulatory efforts may not sufficiently protect this population, even in a state with some of the most stringent heat laws.

It is notable that participants in our study brought up experiences with ‘non-chemical’ stressors such as racism and discrimination, fear of family separation, lack of healthcare, and lack of control and individual autonomy to decide whether to engage in agricultural work without prompting from our study team. Questions in our semi-structured interview guide related primarily to participants’ experiences with climate change and what they would like the general public to know about their work, and we did not ask them specifically about access to healthcare, experiences with law enforcement, discrimination, or similar topics. That these themes emerged organically from study participants underscores how ever-present many of these psychosocial stressors are in farmworkers’ lives; further, efforts to improve climate resiliency and farmworker wellbeing must consider interconnected environmental, occupational, residential, situational, and psychosocial threats to health.

Agricultural workers are and will continue to be one of the groups most vulnerable to the impacts of climate change, and this work highlights some farmworkers’ experiences with climate change in their own words. However, it is important to note that the primary intention of this work was to conduct a digital storytelling project to humanize this essential population and not to conduct an extensive qualitative analysis. Future work should consider examining farmworkers’ suggestions for improving climate resiliency and adaptation, including policy changes and interventions.

## 5 Conclusion

This qualitative digital storytelling project highlighted farmworkers’ experiences with climate change, including health impacts and their limited perceived ability to protect themselves due to situational factors such as limited access to shade or ability to take breaks at work. Participants also reported various psychosocial stressors such as discrimination, lack of access to healthcare, and fear of deportation and family separation that will continue to exacerbate the impacts of these climate-intensified exposures. Farmworkers are one of the groups most vulnerable to the impacts of climate change, and exposure to extreme heat, wildfire smoke, and climate disasters will continue to worsen in the coming decades. Findings from this work underscore the importance of shifting the burden of climate resiliency from individuals to systemic workplace, residential, and community interventions to protect and improve the health and wellbeing of this essential population.

## Author statement

All authors have read the manuscript, accept responsibility for the contents, and are in agreement that the work is ready for submission to the journal. The authors have no potential competing financial interest regarding this manuscript. This manuscript presents original research, and has not been previously published and has not been submitted elsewhere for publication.

## Funding

This work was supported by the Food and Farms Communication Fund at the Greater Kansas City Community Foundation.

## CRediT authorship contribution statement

**Carly Hyland:** Writing – review & editing, Writing – original draft, Supervision, Resources, Project administration, Investigation, Funding acquisition, Conceptualization. **Delmy Flores:** Writing – review & editing, Investigation. **Grace Augusto:** Writing – review & editing, Investigation. **Irene Ruiz:** Writing – review & editing, Investigation, Funding acquisition, Conceptualization. **Marielena Vega:** Writing – review & editing, Investigation. **Rulon Wood:** Writing – review & editing, Supervision, Resources, Funding acquisition, Conceptualization.

## Declaration of competing interest

The authors declare that they have no known competing financial interests or personal relationships that could have appeared to influence the work reported in this paper.
